# Income in Multiple Sclerosis Patients with Different Disease Phenotypes

**DOI:** 10.1371/journal.pone.0169460

**Published:** 2017-01-12

**Authors:** Andrius Kavaliunas, Ali Manouchehrinia, Virginija Danylaite Karrenbauer, Hanna Gyllensten, Anna Glaser, Kristina Alexanderson, Jan Hillert

**Affiliations:** 1 Department of Clinical Neuroscience, Karolinska Institutet, Stockholm, Sweden; 2 Department of Neurology, Karolinska University Hospital, Stockholm, Sweden; Heinrich-Heine-Universitat Dusseldorf, GERMANY

## Abstract

**Background:**

Multiple sclerosis (MS) is a disease with profound heterogeneity in clinical course.

**Objective:**

To analyze sources and levels of income among MS patients in relation to disease phenotype with a special focus on identifying differences/similarities between primary progressive MS (PPMS) and secondary progressive MS (SPMS).

**Methods:**

A total of 6890 MS patients aged 21−64 years and living in Sweden in 2010 were identified for this cross-sectional study. Descriptive statistics, logistic, truncated linear, and zero-inflated negative binomial regression models were used to estimate differences in income between SPMS, PPMS and relapsing-remitting MS (RRMS) patients.

**Results:**

RRMS patients earned almost twice as much as PPMS and SPMS patients (on average SEK 204,500, SEK 114,500, and SEK 79,800 in 2010, respectively). The difference in earnings between PPMS and SPMS was not statistically significant when analyzed with multivariable regression. The estimated odds ratio for PPMS patients to have income from earnings was not significantly different from SPMS patients (95% CI 0.98 to 1.59). PPMS and RRMS patients were less likely to receive benefits when compared to SPMS patients (by 6% and 27% lower, respectively).

**Conclusion:**

Our findings argue for similarities between PPMS and SPMS and highlight the socioeconomic importance of preventing RRMS patients convert to SPMS.

## Introduction

Multiple sclerosis (MS) is one of the most common causes of neurological disability in young adults, having a significant socioeconomic impact for patients, which is further amplified by the relatively early age of MS onset [1–3]. The clinical course of MS may indeed follow a variable pattern [4], but three major variants of MS have gained general acceptance, namely relapsing-remitting MS (RRMS), primary progressive MS (PPMS) and secondary progressive MS (SPMS). The most common presentation of MS is RRMS which has an onset in young adulthood and runs an irregular course. In contrast, PPMS has a later onset and a generally predictable course, as PPMS patients progress continuously [5]. Eventually, most patients with RRMS convert into SPMS. The annual conversion rate into SPMS is approximately 2.5% [6]. Within 20–30 years the majority of RRMS patients have converted to SPMS [7].

However, recently, this division of MS phenotypes has between challenged and alternative classification of MS phenotypes and subtypes has been suggested, based on a combined consideration of disease activity and progression. It emphasizes the importance of accurate descriptions of MS phenotypes for communication, prognostication, design and recruitment to clinical trials as well as for treatment decisions [8]. Although social determinants are not suggested in clinical course descriptions of MS [9], it is a possibility to explore these parameters further as potentially novel outcomes in a variety of research questions in MS [10]. Such parameters, in particular income and educational level, can contribute to the understanding of disease distribution and severity, as well as influence clinicians and public health decisions. Socioeconomic factors are closely related to each other as they represent certain dependent interactions and there is a need to improve our understanding of the ways they affect health and disease [11].

The aim of this study was to use socioeconomic data, i.e. sources and levels of income among MS patients, as a tool to address differences between MS phenotypes, with a special focus on differences/similarities between PPMS and SPMS.

## Materials and Methods

A cross-sectional population-based study was conducted, linking data from the following two sources:

1. The nationwide *Swedish Multiple Sclerosis Register* (SMSreg) was used to obtain information about individuals diagnosed with MS, age at MS onset, and the clinical course. SMSreg runs on government funding and is used in all Swedish neurology departments. Currently the SMSreg includes data on 14,500 of Sweden´s estimated 17,500 prevalent patients with MS [12, 13].
2. The *Longitudinal Integration Database for Health Insurance and Labor Market Studies* (LISA) held by Statistics Sweden was used to obtain information on socio-demographic variables (age, sex, living region in the country, family composition, type of living area, country of birth, education) and annual amounts of six sources of incomes: earnings, disability pension, sickness absence, disability allowance, unemployment compensation, and social assistance in Swedish Crowns (SEK) for individuals aged 21−64 and living in Sweden during 2010 [14].

The unique personal identification number assigned to all residents in Sweden was used to conduct the linkage of data.

Our study population has been described in detail previously [10]. In short, these were 7929 MS patients, aged 21−64 years and living in Sweden in 2010 that had at least one clinical visit during a five year period (2008−2012). In this study we had to exclude 1039 patients additionally due to missing information regarding MS phenotype. In total, 6890 MS patients were included in the final analysis with either RRMS, PPMS or SPMS (progressed before 2010) phenotype.

### Statistical analyses

**Fig 1:**
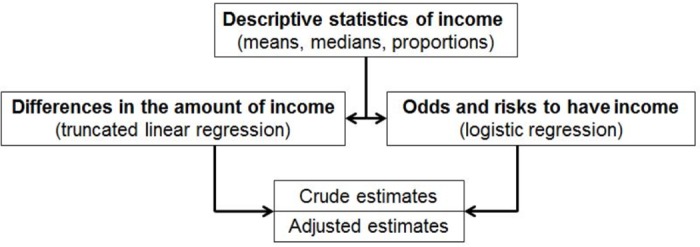
Analysis approach.

We analyzed income data separately for ‘earnings’ and the sum of the other five sources of incomes (disability pension, sickness absence, disability allowance, unemployment compensation, and social assistance): ‘benefits’ [14, 15]. Our statistical analysis approach is shown in Fig 1. Both, income of the patients and MS phenotype, were assessed in 2010. Descriptive statistics were used to describe, in absolute and relative terms, earnings and benefits. Student's t-test was used to compare continuous variables (income data) between PPMS and SPMS patient groups. For the categorical variables, a Chi-square test was used; for medians, a Kruskal-Wallis test was used.

Multivariable regression model was used to analyze income (dependent variable) among MS patients with different phenotypes (categorical independent variable). The model was adjusted for age, disease duration, sex, geographical region, family composition, type of living area, country of birth and education (and scores of the Expanded Disability Status Scale (EDSS), additionally). The regression coefficients (presented in hundreds in the tables) were interpreted as the estimates of income in SEK (the average exchange rate of EUR 1 = SEK 9.5 and USD 1 = 7.2 SEK in 2010 [16]). The truncated linear regression models with left truncation for having no earnings and no benefits were used to estimate the differences between RRMS, PPMS, and SPMS patients regarding their income for those who had it. Zero-inflated negative binomial regression was used to acquire incidence rate ratios (IRR) and express relative differences in income between the phenotypes.

Multivariable logistic regression analysis was performed to estimate the probability for having earnings and benefits. The estimates were interpreted as odds ratios (OR) with 95% confidence intervals (CI). As our outcomes were rather common (e.g., 92.3% of the SPMS had income from benefits), to avoid the possible misleading interpretation of ORs, we converted these estimates into corrected prevalence ratios (PR) using the formula below (1) [17, 18]:

$$PR=\frac{OR}{\left(1-p_{ref}\right)+\left(p_{ref}\times OR\right)}$$ (1)

Where *p*_*ref*_ indicates the probability of the outcome of interest (earnings or benefits) in the reference group. To highlight the difference between SPMS and PPMS patients, SPMS was chosen as a reference group. Differences were defined as statistically significant for p values lower than 0.05. The coefficient of determination values (adjusted R^2^ for the linear regression and pseudo R^2^ for the logistic regression) were used to assess how regression models explain variability.

The project was approved by the Regional Ethical Review Board of Stockholm.

## Results

### Patients´ characteristics

Of the 6890 patients, 5018 (72.8%) were RRMS, 1410 (20.5%) were SPMS, and 462 (6.7%) were PPMS patients. Among these, 4965 (72.1%) were women and 1925 (27.9%) were men. The mean age of the patients was 44.6 ± 10.9 years and mean age at onset of the disease was 32.0 ± 9.7 years (Table 1; additional descriptive data can be found in S1 Table).

**Table 1:** Descriptive data of the study population of MS patients by phenotype.

Patients´ characteristics	All selected (N = 6890)	Phenotype			P value*
		RRMS (n = 5018)	SPMS (n = 1410)	PPMS (n = 462)	
**Gender**					
Men	1925(27.9%)	1293(25.8%)	430 (30.5%)	202 (43.7%)	<0.001
Women	4965 (72.1%)	3725 (74.2%)	980 (69.5%)	260 (56.3%)	
**Age** (mean, ±SD)	44.6±10.9	41.4±10.2	53.1±8.0	53.2±8.0	0.72
**Age at MS onset** (mean, ±SD)	32.0±9.7	31.5±9.4	31.0±9.4	40.6±9.4	<0.001
**EDSS** (median)	3	2	6	5	<0.001
**Earnings**					
Number (%) of >0	4960 (72.0%)	4137 (82.4%)	591 (41.9%)	232 (50.2%)	0.002
Mean (in SEK 100)	1730	2045	798	1145	<0.001
Median (in SEK 100)	1421	1973	0	6	<0.001
**Sum of benefits**					
Number (%) of >0	4375 (63.5%)	2687 (53.5%)	1301 (92.3%)	387 (83.8%)	<0.001
Mean (in SEK 100)	626	424	1201	1074	<0.001
Median (in SEK 100)	420	60	1253	1186	0.003

* p value for SPMS and PPMS comparison (Chi-square test, Student's t-test or Kruskal-Wallis test)

Overall, 72.0% of the patients had income from earnings and 63.5% from benefits. These proportions, as well as means and medians of earnings and benefits were significantly different when comparing PPMS and SPMS patients. For example, SPMS patients had the lowest proportion with income from earnings (41.9%); the mean (SEK 79,800) and median (SEK 0) of their earnings were also the lowest compared to PPMS and RRMS patients. Also SPMS patients had the highest proportion to receive income from benefits (92.3%), and the highest mean (SEK 120,100) and median (SEK 125,300) of benefits. RRMS patients earned almost twice as much as PPMS and SPMS patients (Table 1). The SPMS and PPMS patients were similar by their distribution in age groups, family composition and mean of social assistance (S1 Table).

**Fig 2:**
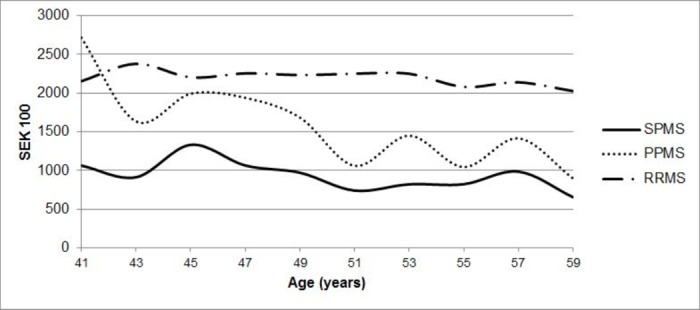
The average of annual earnings of the patients by MS phenotype and age.

As there were only a few relatively young PPMS and SPMS patients, we selected only those of 40–60 years to graphically illustrate the average of annual earnings by age for the different MS phenotypes (Fig 2). It is clearly seen, that in this age interval RRMS and SPMS patients’ earnings were very different at any year (the overall mean among MS patients of 40–60 years was SEK 221,857 and SEK 88,782 for RRMS and SPMS patients, respectively), while PPMS patients´ earnings with age became more similar to those of SPMS patients (the mean among MS patients of 40–60 years was SEK 130,831).

### Combined annual income

**Fig 3:**
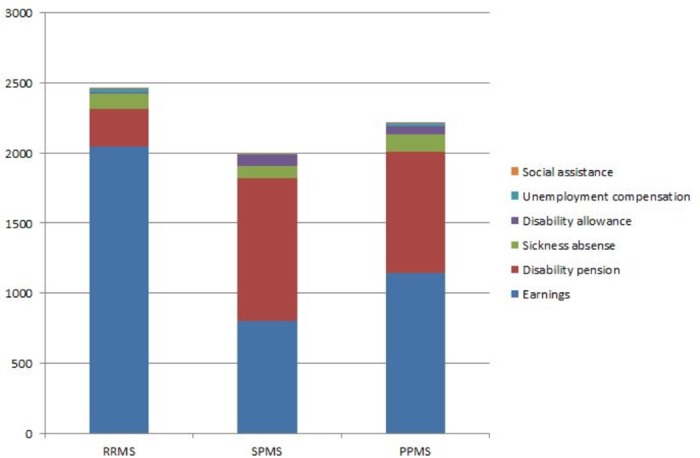
Average annual income of the patients by MS phenotype.

The combined average income of MS patients is shown in Fig 3. SPMS patients had the lowest average of the combined average income, SEK 199,800, but the combined average income among PPMS patients was only 11% higher, SEK 221,800. Earnings were the main source of income for RRMS and PPMS patients and comprised 83% and 52% of total annual income respectively. However, this difference in percentages was smaller between PPMS and SPMS patients (52% and 40%, respectively) than between PPMS and RRMS patients (52% and 83%, respectively). Disability pension was the main source of income among the SPMS patients.

### Differences in the amount of income

Results from the unadjusted and adjusted truncated linear regression analysis (with left truncation for zero values) for the level of income and relative differences by the phenotypes are shown in Table 2. Although PPMS patients had SEK 26,200 (16%) more annual income from earnings and SEK 2600 (1%) less from benefits than SPMS patients, on average, these differences were not statistically significant (95% CI -1600 to 54,000 and -8700 to 3600, respectively). The RRMS patients were very different from the other groups, as they earned significantly more (SEK 75,000 more than SPMS patients; 95% CI 57,900 to 92,100; or 42% more than SPMS patients; IRR = 1.42, 95% CI 1.31 to 1.54)) and received less benefits (SEK 33,500 less than SPMS patients; 95% CI -37,600 to -29,500; or 27% less than SPMS patients, IRR = 0.73, 95% CI 0.69 to 0.77).

**Table 2:** Differences in income in 2010 among MS patients by phenotype.

Phenotype	Earnings			Benefits		
	Crude coefficient (95% CI)	Adjusted coefficient (95% CI)*	Adjusted IRR(95 CI)*	Crude coefficient (95% CI)	Adjusted coefficient (95% CI)*	Adjusted IRR(95 CI)*
**SPMS**	Reference	Reference	Reference	Reference	Reference	Reference
**PPMS**	376 (87 to 665)	262 (-16 to 540)	1.16 (1.02 to 1.32)	-20 (-81 to 41)	-26 (-87 to 36)	0.99 (0.90 to 1.08)
**RRMS**	576 (412 to 714)	750 (579 to 921)	1.42 (1.31 to 1.54)	-511 (-547 to -475)	-335 (-376 to -295)	0.73 (0.69 to 0.77)
Adjusted R^2^	0.01	0.15		0.17	0.24	

Coefficients in the table presented in hundreds and are estimates of differences in income in SEK for PPMS and RRMS when compared to SPMS.

* Adjusted for age, age-squared, disease duration, gender, geographical region, family composition, type of living area, country of birth, education

Unadjusted coefficients, containing only categorized phenotype in the model, resulted in very low adjusted R-squared values, for example 0.01 for the explained variability of earnings, showing that other factors, like those included in the adjusted models (particularly gender and education), have a much stronger impact on the income of MS patients.

### Odds and prevalence ratios for having income

As a part of the study population was excluded due to abundance of zero values in a previous regression analysis, we also conducted a logistic regression for the odds of having different types of income among MS patients. Table 3 shows the results of the multivariable logistic regression analysis presented as ORs for having earnings and benefits for MS patients by phenotypes. The estimated OR for having earnings among RRMS compared to SPMS was 3.98 (95% CI 3.39 to 4.66), while the estimated OR for PPMS was not significantly different from SPMS (95% CI 0.98 to 1.59). Both PPMS and RRMS had lower odds of receiving benefits when compared to SPMS patients (0.55 and 0.17 respectively).

**Table 3:** Odds ratios with corrected prevalence ratios for having income among MS patients by phenotype.

Phenotype	Earnings (>0)		Benefits (>0)	
	OR*(95% CI)	Corrected prevalence ratio(95% CI)	OR*(95% CI)	Corrected prevalence ratio (95% CI)
**SPMS**	Reference	Reference	Reference	Reference
**PPMS**	1.25 (0.98 to 1.59)	1.13 (0.99 to 1.27)	0.55 (0.39 to 0.76)	0.94 (0.89 to 0.98)
**RRMS**	3.98 (3.39 to 4.66)	1.77 (1.69 to 1.84)	0.17 (0.14 to 0.22)	0.73 (0.68 to 0.79)
Pseudo R^2^	0.21		0.19	

* Adjusted for age, age-squared, disease duration, gender, geographical region, family composition, type of living area, country of birth, education.

To avoid the distortion effect of ORs, we converted these estimates into prevalence ratios, for example, the corrected prevalence ratio for having earnings among RRMS patients when compared to SPMS patients was 1.77 (95% CI 1.69 to 1.84). In this analysis, the risk of receiving benefits was lowered only by 6% for PPMS when compared to SPMS, but 27% lower for RRMS patients. Thus PPMS and RRMS were less likely to receive benefits when compared to SPMS patients (corrected prevalence ratios were 0.94 (95% CI 0.89 to 0.98) and 0.73 (95% CI 0.68 to 0.79), respectively).

### Additional adjustments for EDSS

In our regression analyses we adjusted the models for a number of covariates, most importantly gender and education. We were also tempted to include disability in the analysis to investigate to what extent it can improve the explained variance. In fact, introducing disability in the form of EDSS turned the disease phenotype parameter insignificant, both in linear and logistic regression models. Explained variance was somewhat better than in previous analyses (Table 2 and Table 3) (for example, adjusted R-squared was 0.18 in a model with EDSS for truncated linear regression for earnings and 0.28 for benefits, while in the similar models without EDSS these values were 0.15 and 0.24, respectively; full results S2 Table), but intriguingly, the majority of the variance remained unexplained.

## Discussion

In this cross-sectional study, based on the SMSreg and a nation-wide registry of socioeconomic data we assessed income among MS patients in relation to different phenotypes of the disease and other factors. We found that PPMS and SPMS patients were rather similar from the perspective of patients´ income. In contrast, RRMS patients proved to have higher earnings and less benefits than the two other groups.

Reversely, the main limitation of this study is that by a cross-sectional design we could only analyze patients´ income at one particular year, whereas a longitudinal study could address the interaction of age and disease progression, and reveal changes in patients´ income. Identification of early predictors of lower income is also of importance and that warrants further studies. Secondly, our truncated regression analysis was based on data from patients who had earnings and received benefits, thus excluding a part of the patients with no income. Our study population comprised of a relatively large proportion of RRMS (72.8%), mainly influenced by the aim to study income among MS patients of working age, thus excluding elder patients who were entitled for retirement (>64 years). The SMSreg has been active since 2001 [13], thus not surprisingly it also contains more information about newly diagnosed patients since its operating date. This overrepresentation might be an issue in studies where clinical course is in itself an interest as an outcome (e.g., treatment exposure and clinical progression), but less likely to be a potential source of selection bias here, as there is no reason to believe that non-participants with other phenotypes (PPMS, SPMS) would have significantly different earnings or social benefits (or other distributions of socioeconomic variables) than described in this study, or that relation between MS phenotype and patients´ income would be somehow different if we had more SPMS and PPMS patients. On the other hand, the high proportion of RRMS patients in a prevalent sample still might be true since: 1) there is growing evidence of treatment benefits in delaying progression [19, 20]; 2) PPMS seems to have decreased in recent years [21]. Nevertheless, the strengths of our study include a large sample and the population-based register approach where information from two databases were linked enabling use of sociodemographic and clinical data of high quality, that allow stratifications by MS phenotype.

It is still unknown why disease progression in MS may sometimes begin *de novo* or, at other times, only after the patient has experienced a series of clinical relapses. This has led to suggestions that PPMS is a distinct form of the disease and data suggest that SPMS is just a later phase of the same underlying illness as RRMS. Altogether, data from currently available natural history series [22] support the notion, that, from a clinical and statistical perspective, SPMS and PPMS are more similar than they are different. The results of the present study also speak in this direction, i.e. supports homogeneity of MS.

Although in our analyses we could detect some statistically significant differences (for example, medians of benefits among PPMS and SPMS patients were SEK 118,600 and SEK 125,300, respectively; P = 0.003), it can well be questioned whether these differences are meaningful for the patients. For instance, a significant P value tells us that we can rule out a null effect (e.g., a difference of 0) with 95% certainty; but, large samples give such a precision that ruling out the null value is not necessarily relevant. The ruling out of the null hypothesis does not guarantee that the effect is far from 0 [23]. In our case, even from the descriptive data, we can see that although the difference in income between PPMS and SPMS patient groups was statistically significant, the real effect size is not economically meaningful (e.g., the difference of the medians of annual income was SEK 600 (≈EUR 63, ≈USD 83) per year. Thus, SPMS and PPMS patients hardly differ numerically. On the contrary RRMS patients were both statistically and economically different from the other two groups (for example, RRMS patients earned almost twice as much as PPMS and SPMS patients).

The traditional course definitions in MS are based on an interplay between two clinical phenomena, relapses and progression. Both of these processes can lead to irreversible disability, raising the question of which is the more important factor in determining the amount of disability [22]. In a recent study we reported, that individuals with severe disability had 59% lower earnings and 92% higher benefits than patients with mild disability [10]. Once we added EDSS to our regression models, the MS phenotype parameter became insignificant, suggesting that EDSS is a more valuable predictor, which strengthens the previously reported correlation between EDSS and both lower earnings and higher benefits. Yet in this study we focused on picturing the financial situation among MS patients in relation to phenotype, rather than prediction, thus giving more attention to the estimates, adjusted for the well-known socio-economic factors.

As RRMS patients comprise the majority and are less affected by disease progression (e.g., median EDSS 2), it may also be of interest how much they differ from healthy controls. In our recent study we reported that in 2010, both MS patients and matched controls from the total population of Sweden received most of their income through earnings followed by income from disability pension and sickness absence. However, MS patients had 15% lower earnings and 33% higher benefits than the controls [15]. The reported means of earnings and benefits among the healthy controls in 2010 were SEK 245,000 and SEK 19,500, respectively, whereas in the present study the respective means for RRMS patients were SEK 204,500 and SEK 42,400, thus showing that overall the average annual income of RRMS patients was slightly lower than healthy controls (SEK 246,900 and SEK 264,500, respectively).

Another interesting comparison can be made by looking at PPMS patients at the onset of disease. At ages 40–41 the average of annual earnings among PPMS patients was even higher than among RRMS patients (SEK 271,550 and SEK 215,500 respectively), which is not surprising, as the mean onset of MS for PPMS patients was 40.6 (± 9.4) years, therefore at this point their earnings were more similar to those of the general population. For example, the average of annual earnings for healthy controls in 45–54 age group was SEK 279,800 [15]. However, this changes dramatically with age, since, with every year, earnings of PPMS patients lower and gradually become more similar to earnings of SPMS patients. Evidently, gender plays an important role in explaining the observed differences, for reasons such as 1) higher proportion of men in the PPMS patient group, 2) these men are just in the beginning of the disease trajectory with less disability compared to SPMS patients at the same age [24], 3) from 4% to 26% difference in salaries between men and women, depending on job type [25]. Therefore, we provide estimates from the regression models adjusted for age, gender, education and other important covariates on which we had data at our disposal. Results from the multivariate regression analyses indicate similarities in income between the PPMS and SPMS patient groups and highlight the difference from RRMS patients.

Altogether, this study contributes with the insight on MS patients´ financial situation and to our knowledge, this is the first study to examine how MS patients´ income is associated with different disease phenotypes.

## Supporting Information

S1 Table Additional descriptive data of the study population. (DOCX)

S2 Table Regression analyses with additional adjustments for EDS. (DOCX)
