# Supplementary material for: Income in Multiple Sclerosis Patients with Different Disease Phenotypes
Source: PLoS One. 2017 Jan 12;12(1):e0169460. doi: 10.1371/journal.pone.0169460 (PMC5231357; doi:10.1371/journal.pone.0169460)
Supplement: S1 Table — (DOCX) [file pone.0169460.s001.docx]

**S1 Table. Additional descriptive data of the study population**

| **Patients’ characteristics** | **All** selected  (N=6890) | **Phenotype** | | | **P value*** |
| --- | --- | --- | --- | --- | --- |
|  |  | **RRMS**  (n=5018) | **SPMS**  (n=1410) | **PPMS**  (n=462) |  |
| **Age groups**  21-24  25-34  35-44  45-54  55-65 | 283 (4.1%)  1300 (18.9%)  2027 (29.4%)  1939 (28.1%)  1341 (19.5%) | 281 (5.6%)  1258 (25.1%)  1709 (34.1%)  1273 (25.4%)  497 (9.9%) | 1 (0.1%)  30 (2.1%)  249 (17.7%)  496 (35.2%)  634 (45.0%) | 1 (0.2%)  12 (2.6%)  69 (14.9%)  170 (36.8%)  210 (45.5%) | 0.47 |
| **Geographical region**  East  South/West  North | 3341 (42.1%)  3011 (38.0%)  1577 (19.9%) | 2312 (43.2%)  1978 (37.0%)  1057 (19.8%) | 390 (42.6%)  342 (37.4%)  183 (20.0%) | 303 (35.7%)  371 (43.8%)  174 (20.5%) | <0.001 |
| **Family composition**  With partner, no children  With partner, with children  Single, no children  Single, with children | 1403 (17.7%)  3036 (38.3%)  2826 (35.6%)  664 (8.4%) | 726 (13.6%)  2357 (44.1%)  1796 (33.6%)  468 (8.8%) | 224 (24.5%)  310 (33.9%)  297 (32.5%)  84 (9.2%) | 256 (30.2)  227 (26.8%)  301 (35.5%)  64 (7.5%) | 0.32 |
| **Type of living area ***  Larger cities  Medium-sized municipalities  Smaller municipalities | 3428 (43.2%)  2559 (32.3%)  1942 (24.5%) | 2324 (43.5%)  1751 (32.7%)  1272 (23.8%) | 410 (44.8%)  257 (33.7%)  248 (27.1%) | 332 (39.2%)  286 (33.7%)  230 (27.1%) | 0.01 |
| **Country of birth**  Sweden  Other Nordic  Other EU-25  Other | 7138 (90.0%)  193 (2.4%)  155 (2.0%)  443 (5.6%) | 4819 (90.1%)  117 (2.2%)  100 (1.9%)  311 (5.8%) | 826 (90.3%)  23 (2.5%)  19 (2.1%)  47 (5.1%) | 763 (90.0%)  26 (3.1%)  18 (2.1%)  41 (4.8%) | 0.16 |
| **Education ***  Lower  Secondary  Higher | 872 (11.0%)  3713 (46.8%)  3344 (42.2%) | 438 (8.2%)  2423 (45.3%)  2486 (46.5%) | 133 (14.5%)  464 (50.7%)  318 (34.8%) | 149 (17.6%)  418 (49.3%)  281 (33.1%) | 0.002 |
| **Disease duration**, years (mean) | 12.6 | 9.9 | 22.1 | 12.6 | <0.001 |
| **Health related benefits**  (mean in SEK 100) | 621.7 | 349.5 | 932.1 | 1232.2 | <0.001 |
| Disability pension | 482.9 | 241.3 | 720.3 | 1019.6 | <0.001 |
| Sickness absence | 110.0 | 102.9 | 177.2 | 136.3 | 0.02 |
| Disability allowance | 28.8 | 5.3 | 34.7 | 76.3 | <0.001 |
| **Benefits related to low income** (mean in SEK 100) | 29.4 | 36.0 | 22.7 | 8.9 | 0.007 |
| Unemployment compensation | 22.3 | 29.5 | 14.6 | 4.2 | <0.001 |
| Social assistance | 7.1 | 6.6 | 8.1 | 4.7 | 0.65 |

* p value for SPMS and PPMS comparison (Chi-square test, Student's t-test or Kruskal-Wallis test)
