# Supplementary material for: Income in Multiple Sclerosis Patients with Different Disease Phenotypes
Source: PLoS One. 2017 Jan 12;12(1):e0169460. doi: 10.1371/journal.pone.0169460 (PMC5231357; doi:10.1371/journal.pone.0169460)
Supplement: S2 Table — (DOCX) [file pone.0169460.s002.docx]

S2 Table. **Regression analyses with additional adjustments for EDSS**

| **Phenotype** | **Coefficients*** | | **IRR**** | | **ORs***** | |
| --- | --- | --- | --- | --- | --- | --- |
|  | Earnings >0 | Benefits >0 | Earnings | Benefits | Earnings >0 | Benefits >0 |
| **SPMS**  **PPMS**  **RRMS** | Reference  122 (-61 to 306)  70 (-81 to 220) | Reference  -16 (-75 to 42)  -41 (-91 to 8) | Reference  1.05 (0.92 to 1.19)  0.96 (0.90 to 1.10) | Reference  0.99 (0.91 to 1.08)  0.97 (0.90 to 1.04) | Reference  1.12 (0.86 to 1.46)  1.06 (0.86 to 1.30) | Reference  0.68 (0.48 to 0.99)  0.84 (0.64 to 1.09) |
| Adjusted R^2^/  Pseudo R^2^ | 0.27 | 0.31 |  |  | 0.28 | 0.24 |

Coefficients in the table presented in hundreds and are estimates of differences in income in SEK for PPMS and RRMS when compared to SPMS.

* adjusted for age, age-squared, disease duration, gender, geographical region, family composition, type of living area, country of birth, education and EDSS; truncated linear regression

** adjusted for age, age-squared, disease duration, gender, geographical region, family composition, type of living area, country of birth, education and EDSS; zero-inflated negative binomial regression

*** adjusted for age, age-squared, disease duration, gender, geographical region, family composition, type of living area, country of birth, education and EDSS; logistic regression
